# The role of AKT and FOXO3 in preventing ovarian toxicity induced by cyclophosphamide

**DOI:** 10.1371/journal.pone.0201136

**Published:** 2018-08-02

**Authors:** Bao-fang Zhang, YaXin Hu, Xinyan Liu, Zhuo Cheng, Yu Lei, YongMei Liu, Xueke Zhao, Mao Mu, Lei Yu, Ming-liang Cheng

**Affiliations:** 1 The First Affiliated Hospital, Soochow University, Suzhou, Jiangsu, China; 2 The Affiliated Hospital, Guizhou Medical University, Guiyang, Guizhou, China; 3 Shandong Institute of Biological Products,Taishan district, Shandong, China; 4 Peking University Health Science Center School of Foundational Education, Beijing,China; University of Alabama at Birmingham, UNITED STATES

## Abstract

Cyclophosphamide (CTX) has immunosuppressive effects and has been wildly used as one anti-cancer drug in clinical. Significant toxicity has been noticed particularly in the reproductive system. CTX promotes the maturation of ovarian follicles, decreases follicular reserve, and ultimately lead to ovarian failure or even premature ovarian failure (POF). The placental extract (HPE) has been shown to have some beneficial impact on reproductive system; however, little is known regarding to the effect of HPE on protecting CTX-induced ovarian injury and the mechanism involved. Whether human placental extracts (HPE) has a protective effect on CTX-induced toxicity on ovarian was studied by using a CTX-induced ovarian injury animal model. The effects of HEP on histopathology, the number of atretic follicles, the weight of the ovary, serum hormone levels, and apoptosis in granulosa cells were studied in mice with CTX or control vehicle. Our results have demonstrated that HPE inhibited p-Rictor, reduced the expression of Bad, Bax and PPAR, and activated Akt and Foxo3a (increased their phosphorylation). Mice treated with HPE showed higher ovarian weight, lower number of atretic follicles, higher serum levels of the hormones E2 and progesterone, and lower apoptosis and serum levels of LH and FSH in granulosa cells, than that in the control animal group. Our data show that ovarian injury can be attenuated by HPE. HPE likely protects follicular granulosa cells from undergoing significant apoptosis and reduce atresia follicle formation, therefore, alleviates CTX-induced ovarian injury.

## Introduction

In premature ovarian failure (POF), the ovaries become dysfunctional or lost, and persistent amenorrhea and sexual atrophy are observed in some affected women before the age of 40[1]. POF can result from hereditary, metabolic, infectious, autoimmune, iatrogenic (radiotherapy and chemotherapy) and other causes[2]. Moreover, patients with hormonal disorders may exhibit vasomotor and psychiatric symptoms, such as a hectic fever with unsteady movement, perspiration, daze, heart palpitations, and decreased libido. The mechanisms involved in POF remain unclear, and clinical treatments, especially chemotherapy, play important roles as iatrogenic factors[3].

Because cyclophosphamide (CTX) is aneconomical and effective therapy, it is widely used in various diseases, including cancer, haematologic diseases, primary angiitis of the central nervous system, chronic inflammatory demyelinating polyneuropathy (CIDP), rheumatoid arthritis, and nephrotic syndrome. However, CTX exerts a toxic effect by causing abnormal DNA base pairings, leading to misstructured and dysfunctional cells and irreversible injury in the ovary [4]. CTX accelerates the maturation of ovarian primordial follicles into mature follicles, reducing ovarian reserves and thereby leading to ovarian failure in young female patients with POF [5]. Recently, the proliferation, development and maturation of ovary germ cells are reported to be regulated by the mechanistic target of rapamycin (mTOR) pathway, in which Rictor, mTORC2, Akt, and Foxo3a play key roles. Based on recent reports, a mouse model of POF was constructed using 4-vinylcyclohexene diepoxide (VCD) [6,7]. In these mice, molecular functions of Rictor and its downstream effector mTORC2 were affected and both of these apoptosis-related proteins were overexpressed. These effects accelerated follicular atresia and apoptosis, leading to the loss of ovarian functions.

Human placental extracts (HPE) perform many functions, such as enhancing immunity, regulating endocrine processes, extending youth, repairing damaged skin, eliminating ageing skin, beautifying skin, restraining inflammation, and performing anti-allergic functions[8]. Thus, in this study, we had the following aims: (i) to explore the influence of HPE on ovarian function through analysing a series of indicators includinghistopathological patterns, number of atretic follicles, weight of the ovary and body, serum hormone levels, and apoptosis in granulosa cells in a mouse model of CTX-induced ovarian injury and (ii) to use Western blot analysis to detect the expression levels of p-Rictor, mTORC2, p-Akt, p-Foxo3a, Bad, Bax, and PPAR to investigate the molecular mechanisms by which HPE alleviates the CTX-induced ovarian injuries.

## Materials and methods

### Animal experiments

Female C57BL mice 36, 8 weeks old, 16 ~ 20g, SPF grade, Institutional and national guidelines for the care and use of animals were followed and all experimental procedures involving animals were approved by the ACWC (Animal Care Welfare Committee) of Guiyang Medical University (Permit Number:1503063). The mice were individually housed in plastic cages at room temperature (22°C) and maintained on an artificial cycle of 12-h light and 12-h dark. Mice were anesthetized and the cisplatin (Cyclophosphamide Baxter 0054-4130-25) was administered intraperitoneally once daily at doses of 1.2 ml/kg for 14 days. Control animals received injections of phosphate buffered saline(PBS) at doses of 1.2 ml/kg, HPE animals received injections of HPE(National medicine permission number H20046260, the drugs were purchased from Hospital) at doses of 1.2 ml/kg. After induction of POF, different doses of HPE (Group CH+++: high dose 2.4 ml/kg, Group CH++: medium dose 1.2 ml/kg and Group CH+: low dose 0.6 ml/kg) were administered intraperitoneally once daily to mice in the treatment groups for 14 days. Control, model and HPE groups were given the same volume of saline (1.2 ml/kg) daily throughout the treatment period. The animals were euthanized and tissues were harvested at day 28 after HPE treatment.

### Sample collection and preparation for pathological evaluation

After the final administration of HPE or saline, mice were fasted for 12 h and then anesthetized with 10% chloral hydrate. Then 5mL blood samples were collected from an artery in the rat abdomen. Blood samples were centrifuged at 3000 r/min and 4°C for 15 min to obtain serum samples. After blood collection, ovary samples were removed and weighed. Then samples were fixed in 4% paraformaldehyde (Sigma-Aldrich) for subsequent paraffin embedding. Ovary specimens were sectioned at 5μm and then stained with hematoxylin and eosin (H&E).

### ELISA assay

Commercially available enzyme-linked immunosorbent assay (ELISA) kits were used to determine serum hormone levels of estrogen (E2), follicle-stimulating hormone (FSH), luteinizing hormone (LH) and progesterone according to the manufacturer’s instructions. Briefly, 100mL of mouse E2 or FSH at concentrations of 8,000, 4,000, 2,000, 1,000, 500, 250, and 125pg/mL or 10, 5, 2.5, 1.25, 0.625, 0.312, and 0.156ng/mL or diluted mouse plasma were added to each antibody percolated microtest wells and incubated for 60min. After 3 times of washing, the HRP-conjugated detection antibodies were added followed by substrate solution. The absorbance was determined at a wavelength of 450nm.

### Flow cytometry analysis

Flow cytometry analysis was performed to measure the apoptotic status of granulosa cell. Briefly, 100 μL cells at 5×10^5^/mL were transferred into 5 mL flow tubes. Annexin V/fluorescein isothiocyanate (FITC) (5 μL) was added, and apoptotic rates were detected based on the fluorescence of Annexin V/FITC with a flow cytometer (Coulter Epics XL; Beckman Coulter, Fullerton, CA, USA).

### Western blotting

The protein concentration of the whole ovary lysate was determined by BCA kit (Pierce, Rockford, IL). Lysates were electrophoresed on a disulfide-reduced 12% SDS PAGE, transferred to Immobilon-P membrane (Millipore Corp., Bedford, MA), probed and stripped followed by re-probing with indicated antibodies, and developed with the enhanced chemiluminescent (ECL) system (Pharmacia Biotech, Piscataway, NJ). The expression of GAPDH protein was used as a loading control. For densitometric analysis of band intensity, a specific band on the ECL-developed film was subjected to densitometric analysis (Adobe Photoshop). The densitometric readings were pooled and averaged from three independent experiments. The background of densitometric reading on the ECL-developed film was subtracted.

### Data analysis

Data from at least three independent duplicates were expressed as mean ± SD. Differences between two groups were analyzed with Student’s t-test. For animal studies, each experimental and control group contained 5 to 8 animals and repeated twice. A p value of < 0.05 was considered statistically significant. All statistical analyses were done with the SPSS 20.0 software program (SPSS Inc, Chicago, IL, USA).

## Results

### HPE protects follicles in POF mice

**Fig 1:**
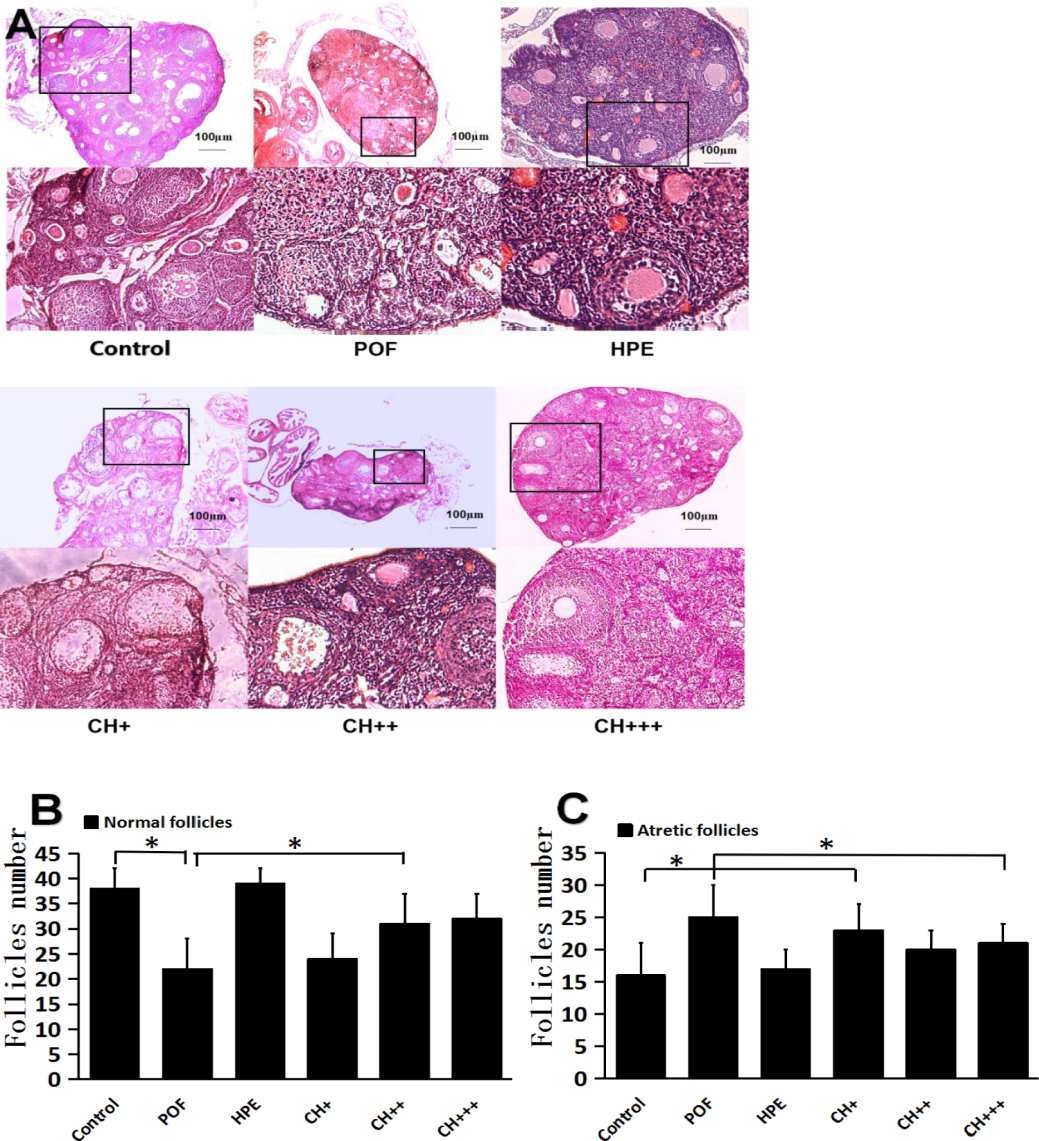
A. Histological comparison of ovarian morphology. B. Comparison of the total number of classified follicles. C. Comparison of the total number of atretic follicles. Compared with the control group, the number of normal follicles in POF, CH +, CH ++ and CH +++ groups was significantly decreased (* P <0.05 vs. the control group); Compared with the POF group, the number of normal follicles in HPE, CH ++ and CH +++ group was significantly increased (* P <0.05 vs. the POF group); Compared with the Control group, the number of atretic follicles in POF and CH + groups was increased significantly (*P <0.05 vs. the control group); Compared with the POF group, the number of atretic follicles in the HPE, CH ++ and CH +++ groups was significantly reduced (*P <0.05 vs. the POF group).

In histopathologically stained sections of ovaries from control mice and HPE mice, large numbers of follicles were observed at various stages of development. In contrast, fewer or no follicles were observed in ovarian sections from POF mice and CH+ mice, which showing larger atretic follicle instead of normal follicles. In ovarian sections from CH++ and CH+++ mice, fewer atretic follicle and mature follicles resembling those in control mice were observed (Fig 1A). The number of follicles (atretic or normal follicles) in each group was counted from 5 sections of each ovary. The results showed a significantly higher number of atretic follicles in the ovaries of the POF group than that in the CH++ and CH+++ groups (Fig 1C). There were fewer normal follicles in the POF group than that in the CH++ and CH+++ groups (Fig 1B and S1B Table).

### HPE induced an increase in serum E2 and progesterone levels and a decrease in serum levels of FSH and LH in POF mice

**Fig 2:**
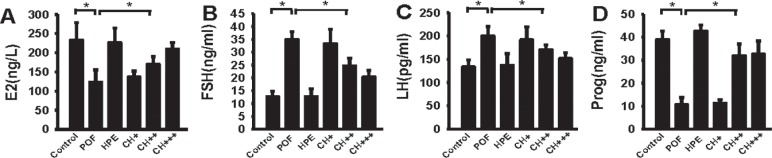
Determination of serum hormone levels. **A.** Compared with the control group, serum levels of E2 was significantly decreased in POF, CH +, CH + + groups (* P <0.05). Compared with the POF group, serum levels of E2 was significantly increased in HPE, CH++ and CH+++ groups (* P <0.05). **B**. Compared with the control group, serum levels of FSH was significantly increased in POF, CH +, CH + + and CH+++groups (* P <0.05). Compared with the POF group, serum levels of FSH was significantly decreased in HPE, CH++ and CH+++ groups (* P <0.05). **C.** Compared with the control group, serum levels of LH were significantly increased in POF, CH + and CH + + groups (* P <0.05). Compared with the POF group, serum levels of FSH was significantly decreased in HPE, CH++ and CH+++ groups (* P <0.05). **D.** Compared with the control group, serum levels of Progesterone was significantly decreased in POF, CH + and CH + + groups (* P <0.05). Compared with the POF group, serum levels of Progesterone were significantly increased in HPE, CH++ and CH+++ groups (* P <0.05).

In order to investigate the levels of sex-related hormones in mice, we analysed the serum levels of the oestradiol E2, FSH, LH and progesterone. The results showed that FSH and LH levels were significantly higher, while E2 and progesterone levels were significantly lower (P <0.05) in the POF group than in the control group. After the mice were treated with a medium or high dose of HPE, serum FSH and LH levels were significantly lower and serum E2 and progesterone levels were significantly higher than that in the POF group (Fig 2). However, there was no significant difference between control and HPE groups (P> 0.05).

### Ovarian and body weight were increased in POF mice treated with medium or high dose of HPE

**Fig 3:**
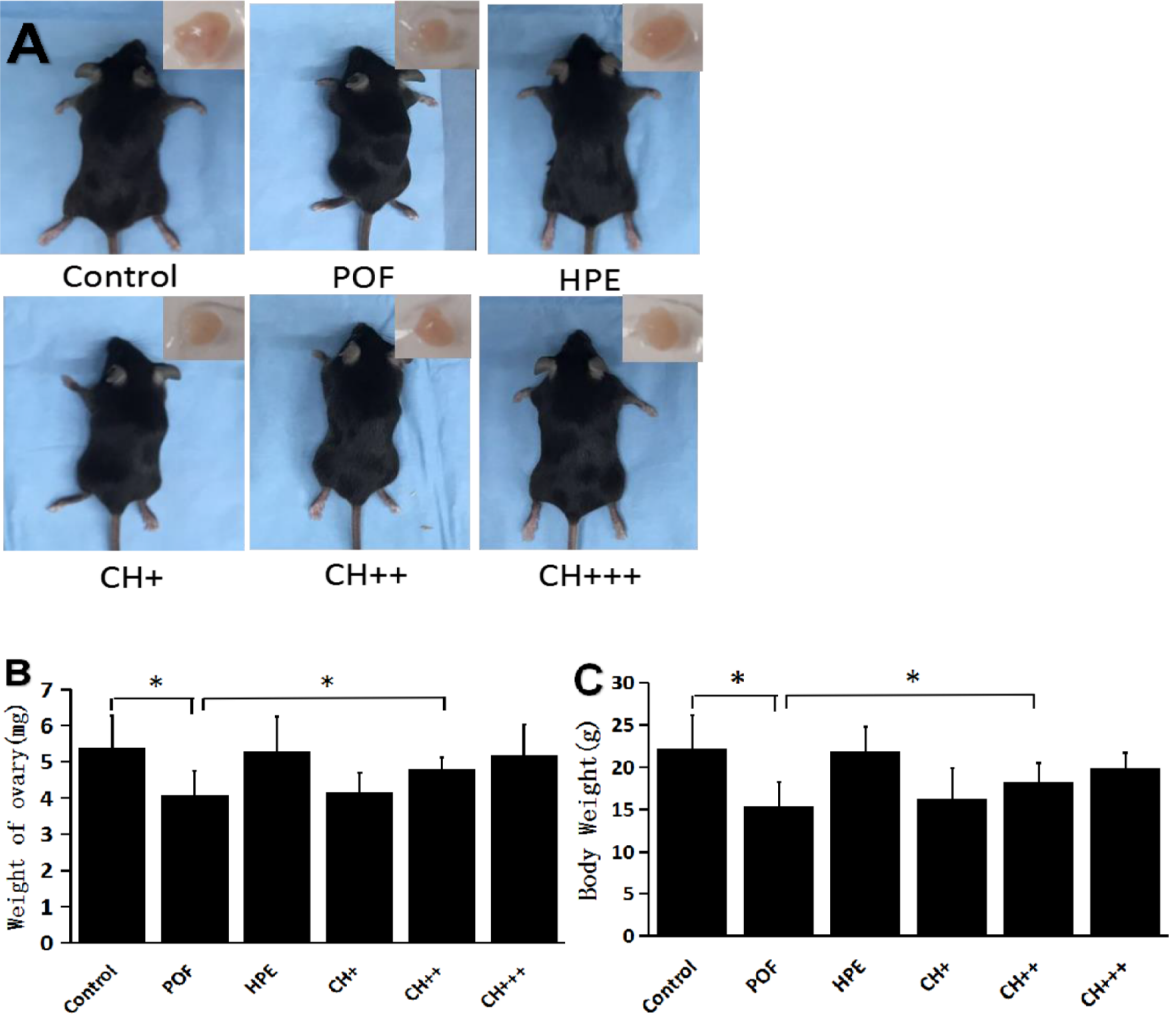
Weight of ovary and body. **A.** Gross morphology of mice and ovaries of each group. **B.** Compared with the control group, the ovarian weight of POF and CH + groups was significantly decreased, (*P<0.05). Compared with the POF group, the ovarian weight of HPE, CH++ and CH+++ groups was significantly increased (*P<0.05). **C.** Compared with the control group, the body weight of POF, CH + and CH++ groups was significantly decreased (*P<0.05), Compared with the POF group, the bodyweight of HPE, CH++ and CH+++ groups was significantly increased.

Body weight and ovarian weight of POF mice were significantly lower than that in the control groups. Following treatment with a medium or high dose of HPE, both body weight and ovarian weight of HPE mice was significantly higher comparing to the POF group. However, there was no difference between the POF and the low dose group(P> 0.05). (Fig 3 and S3B and S3C Table).

### HPE treatment inhibits apoptosis in mouse ovarian granulosa cells

**Fig 4:**
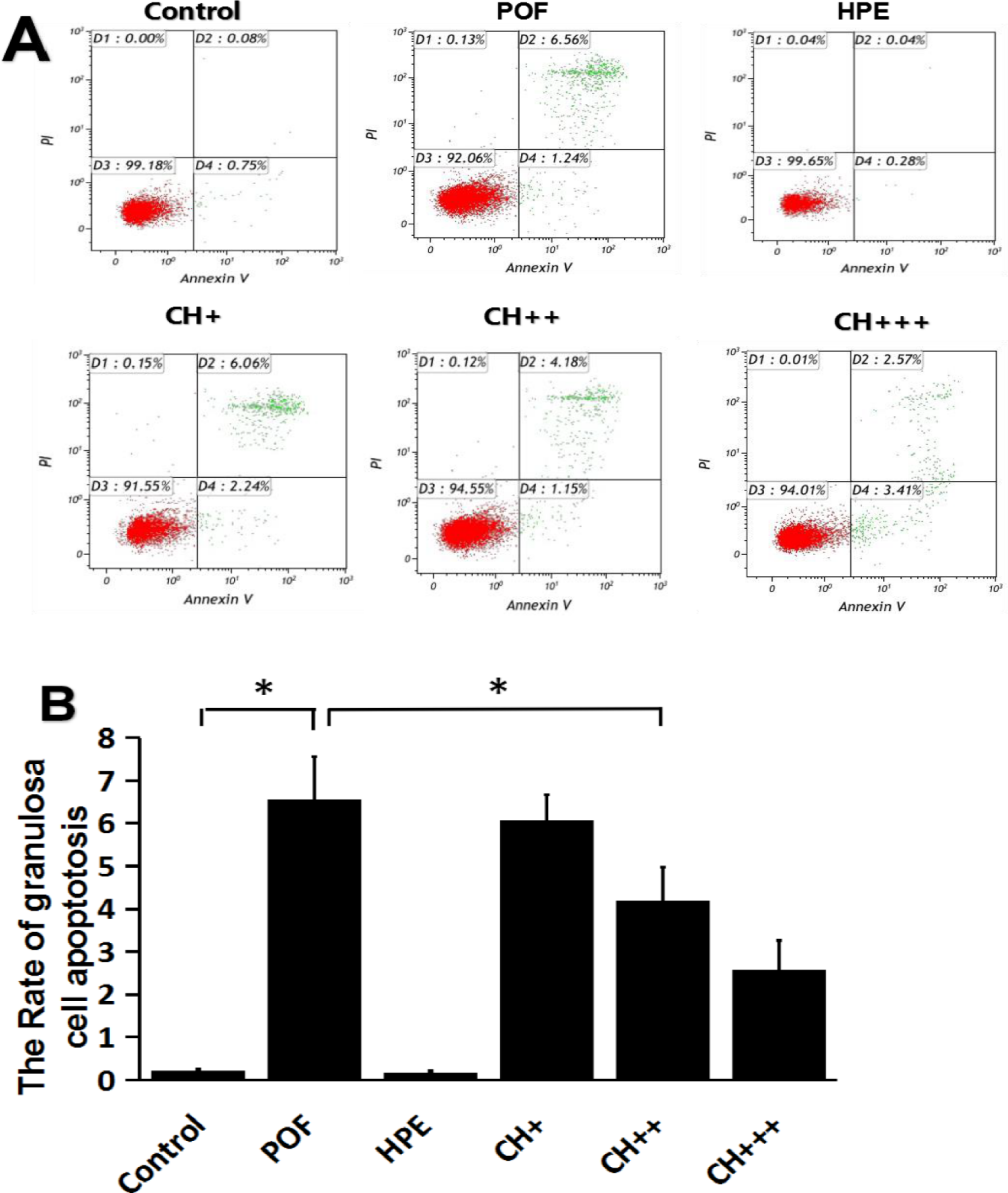
Apoptosis of granulosa cell. **A.** Flow cytometry analysis was performed to measure the apoptotic status of granulosa cell. **B.** Compared with control group, the apoptotic rate of ovarian granulosa cells in POF, CH +, CH ++ and CH +++ groups was significantly higher (*P<0.05); Compared with POF group, the sum of apoptotic rate of ovarian granulosa cells in HPE, CH ++ and CH +++ groups was significantly lower (*P<0.05).

Atretic follicles formation is associated with excessive apoptosis in ovarian granulosa cells. To better understand the role of apoptosis in the formation of atretic follicles, we examined the percentage of cells undergoing apoptosis in ovarian granulosa cells with different treatment by flow cytometry (Fig 4A). The results showed that the rate of apoptosis was significantly higher in ovarian granulosa cells in the POF group comparing to the control group. After mice were treated with a medium or high dose of HPE, the rate of apoptosis was significantly lower in mouse ovarian granulosa cells comparing to the POF group (P <0.05, Fig 4B and S4B Table). These results indicated that the alleviating effect of HPE on POF may be mediated through inhibiting the excessive apoptosis of ovarian granulosa cells.

### HPE inhibited the protein expression of p-Rictor, Bad, Bax, and PPAR and promoted the protein expression of p-AKT and p-Foxo3a in a mouse model of POF

**Fig 5:**
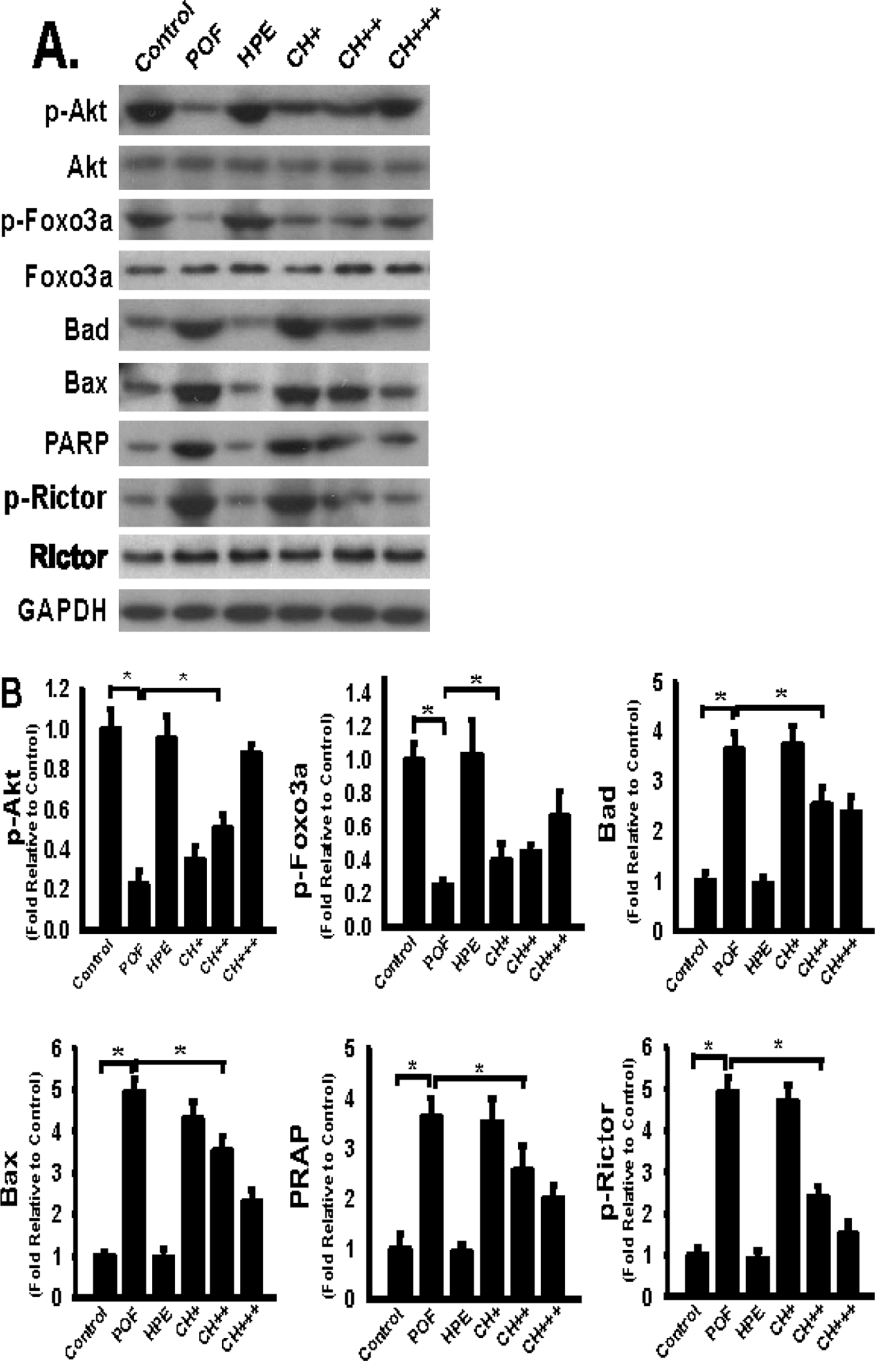
HPE inhibited protein expression of p-Rictor, bad, bax, PPAR and promoted protein expression of p-AKT and p-Foxo3a in POF model mice. **A.** Ovaries were harvested at day 1 after the final administration of HPE or saline, equivalent amount of whole ovary detergent lysates was western blotted with indicated antibodies. Per lane represents individual animals (5–8 animals per group). **B.** Compared with control group, the level of p-AKT and p-Foxo3a in POF group was significantly lower, the level of p-Rictor, Bad, Bax, PPAR in POF group was significantly higher, (*P<0.05). Compared with POF group, the level of p-Rictor, Bad, Bax, PPAR, p-AKT and p-Foxo3a in CH+, CH ++ and CH +++ groups was significantly higher (*P<0.05). Data were pooled and represented as mean ± SD, n = 5–8 animals per group.

The Rictor/mTORC2/Akt/Foxo3a signalling pathway plays a vital role in apoptosis and follicular atresia formation. We next examined the key signalling molecules involved in this pathway. The results showed that the POF group exhibit higher levels of p-Rictor, Bad, Bax and PPAR, but lower levels of p-AKT and p-Foxo3a in the ovary. Remarkably, HPE treatment of the POF mouse showed significantly reduced levels of p-Rictor, Bad, Bax and PPAR, and increased levels of p-AKT and p-Foxo3a (Fig 5).

## Discussion

The mechanisms involved in POF remain unclear. A variety of causes, such as a decrease in the number of original follicles, increased and accelerated follicular atresia, and disordered follicular maturation, can lead to POF [9,10]. Because CTX exerts strong toxic effects in the gonads, its medical uses often lead to POF. The use of CTX is a common contributor to iatrogenic effects and can cause conditions including menstrual disorders, amenorrhea, infertility, and decreased libido. POF decreases each follicular stage and is associated with interstitial fibrosis and necrosis in addition to endocrine changes that can decrease the serum hormone levels of E2 and progesterone and increase the serum hormone levels of LH and FSH [11,12].

It has been reported that the mTOR signalling pathway regulates the growth and development of follicles. mTOR is a highly conserved Ser/Thr protein kinase that contains two unique functional domains for mTORC1 and mTORC2[13–17]. MTORC1 consists of mLST8 and Raptor and regulates cell growth, proliferation, and metabolism. Activated MTORC1 can initiate the downstream phosphorylation of S6K1 and 4E-BP1, thereby promoting the production of egg cells and protein synthesis. MTORC2 is composed of mSIN1 and mLST8 and its core functional component, Rictor. Rictor regulates the functions of its downstream targets Akt, Rho, Rac and Cdc42, which themselves regulate egg cell survival and the construction of the cytoskeleton [18–21].

At present, the upstream and downstream cytokine functions of mTORC1 have been extensively studied. However, the biological functions of mTORC2 are not yet completely understood. Researchers set up a mouse model of POF using 4-vinylcyclohexene diepoxide (VCD) and explored its effects on a variety of factors (e.g., phosphorylation in response to Rictor overexpression, the phosphorylation of Akt and the inhibition of Foxo3a expression) and on Rictor and its downstream mTORC2 signalling molecules (e.g., the Rictor/mTORC2/Akt/Foxo3a signalling axis) [22–25]. However, some proteins associated with apoptosis (e.g., Bad, Bax and PARP) were overexpressed, resulting in the acceleration of follicular atresia and an increase in apoptosis, eventuallyleading to the loss of ovarian physiological functions. In mice, CTX induced POF presents similar results, including a decrease in the expression in the anti-apoptotic protein Bcl-2 and an increase in the expression of the pro-apoptotic protein Bax. These effects together induced apoptosis in follicular granulocyte cells and increased follicular atresia [26].

Currently, hormone replacement therapy is widely used to treat POF even though it causes a variety of adverse reactions andside effects, and their long-term safety and efficacy should be improved. Thus, development of new treatments that aim at restoring ovarian function and improving patient life quality remains a challenge for domestic and international researchers [27].

HPE is a low molecular weight peptide with biological activity that is extracted from human placenta. HPE contains more than 8000 kinds of nutrients and the molecular weight is only 3000 daltons. HPE has few side effects and can be absorbed by the body via intramuscular or intravenous infusion. In vitro experiments showed that HPE accelerates osteoblast mineralization to promote fracture healing [28]. HPE has also been shown to induce colon cancer cell apoptosis, block cell cycle progression, and inhibit cell proliferation and migration, all of which may inhibit tumour cell proliferation [29].

In this study, we use a mouse model in which POF was induced by CTX. We show that this model presents CTX induced ovarian dysfunction which mimics the outcomes present by chemotherapy drugs used in clinical trials. We analysed the pathology of mice ovaries, counted atresia follicles, evaluated ovarian weight, determined serum sex hormone levels, measured ovarian granulosa cell apoptosis, and explored the pathogenesis caused by CTX-induced POF. Moreover, we treated mice with CTX-induced POF with different doses (e.g., high, medium and low doses) of HPE to evaluate its curative effects and explore the underlying molecular mechanisms.

The results showed that CTX led to a decrease in body weight and ovarian atrophy. In the treated mice, pathological examinations showed that the number of normal follicles was significantly lower, the number of atretic follicles was significantly higher, the rate of apoptosis in ovarian granulosa cells was higher, serum E2 and progesterone levels were lower, and FSH and LH levels were higher, altogether resulting in a phenotype similar to that of typical POF. In the Rictor/mTORC2/Akt/Foxo3a signalling axis, P-Rictor, Bad, Bax, and PPAR were upregulated, whereas p-Akt and p-Foxo3a were downregulated. Dysregulation of Rictor/mTORC2/Akt/Foxo3a signalling axis could result in excessive follicular apoptosis and may therefore represent an important mechanism of POF.

A comparison between the control group and the groups treated with high and medium doses of HPE for 14 days showed that treatment with HPE increased weight gain in both the ovary and the body, reduced the number of atretic follicles, increased the serum levels of the hormones E2 and progesterone, reduced the serum levels of the hormones LH and FSH, and decreased apoptosis in granulosa cells. We further demonstrated that HPE treatment downregulated p-Rictor, Bad, Bax, PPAR and upregulated p-Akt and p-Foxo3a. These effects together may protect follicular granulosa cells from undergoing apoptosis, preventfollicular atresia, and ultimately alleviate symptons of CTX-induced ovarian injury.

Our results provide convincing evidence that the mouse POF model can be alleviated by HPE in a dose dependent manner, and thus will be useful to relieve symptoms in POF. This study also has some limitations. First, although symptoms of POF model was successfully alleviated by HPE treatment, the number of mice used for this study was small. This is an acute animal model relative to human diseases, and this is only one of the many models that can induce ovarian injury. This is a toxic model and its conclusion may not be used to other situation and more studies are needed to understand if HPE will benefit on the other types of ovarian injury. Second, HPE is a complex extract which providing large range of effective ingredients. More studies will be needed to identify the effective ingredents. Third, only a few biological studies were performed. Furthermore, although POF-associated markers were evaluated in this study, necessary biological studies can be added according to the purpose and direction of future experiments. It should be cautious to apply the findings directly to human and clinical usage without further studies or human studies.

## Supporting information

S1 File (RAR)
