# Correction: The role of AKT and FOXO3 in preventing ovarian toxicity induced by cyclophosphamide

**DOI:** 10.1371/journal.pone.0215616

**Published:** 2019-04-15

**Authors:** Bao-fang Zhang, YaXin Hu, Xinyan Liu, Zhuo Cheng, Yu Lei, YongMei Liu, Xueke Zhao, Mao Mu, Lei Yu, Ming-liang Cheng

There is an error is affiliation 1 for author Bao-fang Zhang. The correct affiliation 1 is: The Second Affiliated Hospital, Soochow University, Suzhou, Jiangsu, China.
